# A Molecular Approach for Detecting Bacteria and Fungi in Healthcare Environment Aerosols: A Systematic Review

**DOI:** 10.3390/ijms25084154

**Published:** 2024-04-09

**Authors:** Jacek Matys, Julia Kensy, Tomasz Gedrange, Ireneusz Zawiślak, Kinga Grzech-Leśniak, Maciej Dobrzyński

**Affiliations:** 1 Oral Surgery Department, Medical University of Wroclaw, 50-425 Wroclaw, Poland; tomasz.gedrange@umw.edu.pl (T.G.); kinga.grzech-lesniak@umw.edu.pl (K.G.-L.); 2 Faculty of Dentistry, Medical University of Wroclaw, 50-425 Wroclaw, Poland; julia.kensy@student.umw.edu.pl; 3 Faculty of Biotechnology and Food Sciences, Wrocław University of Environmental and Life Sciences, 37 Chełmońskiego Str., 51-630 Wrocław, Poland; ireneusz.zawislak@upwr.edu.pl; 4 Department of Periodontics, School of Dentistry, Virginia Commonwealth University, Richmond, VA 23284, USA; 5 Department of Pediatric Dentistry and Preclinical Dentistry, Wroclaw Medical University, Krakowska 26, 50-425 Wrocław, Poland; maciej.dobrzynski@umw.edu.pl

**Keywords:** aerosol, bacteria, fungi, molecular methods, PCR, 16S rRNA

## Abstract

Molecular methods have become integral to microbiological research for microbial identification. This literature review focuses on the application of molecular methods in examining airborne bacteria and fungi in healthcare facilities. In January 2024, a comprehensive electronic search was carried out in esteemed databases including PubMed, Web of Science, and Scopus, employing carefully selected keywords such as ((bacteria) OR (virus) OR (fungi)) AND (aerosol) AND ((hospital) OR (healthcare) OR (dental office)) AND ((molecular) OR (PCR) OR (NGS) OR (RNA) OR (DNA) OR (metagenomic) OR (microarray)), following the PRISMA protocol. The review specifically targets healthcare environments with elevated concentrations of pathogenic bacteria. A total of 487 articles were initially identified, but only 13 met the inclusion criteria and were included in the review. The study disclosed that the prevalent molecular methodology for appraising aerosol quality encompassed the utilization of the PCR method, incorporating either 16S rRNA (bacteria) or 18S rRNA (fungi) amplification techniques. Notably, five diverse molecular techniques, specifically PFGE, DGGE, SBT, LAMP, and DNA hybridization methods, were implemented in five distinct studies. These molecular tests exhibited superior capabilities compared to traditional bacterial and fungal cultures, providing precise strain identification. Additionally, the molecular methods allowed the detection of gene sequences associated with antibiotic resistance. In conclusion, molecular testing offers significant advantages over classical microbiological culture, providing more comprehensive information.

## 1. Introduction

An aerosol is characterized as a mixture of solid or liquid particles suspended in a gas, such as air, with varying dimensions and origins [1]. Notably, a significant proportion of particles in aerosols consists of bacteria and has drawn attention from researchers due to their potential to transmit infections. Healthcare facilities in particular merit special consideration due to heightened exposure to bacteria-contaminated aerosols compared to other environments [2,3,4,5,6]. Concerns about nosocomial infections, including the airborne transmission of pathogenic bacterial strains, have been highlighted in healthcare settings [7]. Scientists have predominantly used culturing techniques to analyze the microbiological content of aerosols, involving the cultivation of bacteria from a sample on specific media (both solid and liquid) to form countable colonies [8,9,10]. However, this method has limitations, including low sensitivity, as not all bacteria can be cultured in the laboratory, leading to an underestimation of overall bacterial diversity. Furthermore, culture techniques have low specificity as they do not provide detailed information on the exact taxonomic origin, genus, or species of bacteria present in the sample [8].

In recent years, advanced molecular methods have been employed for the molecular-level study of bacterial communities [11,12]. These techniques focus on the extraction and analysis of bacterial genetic material, eliminating the need for culturing. This approach enhances our understanding of the oral microbiota, encompassing both cultivable and non-cultivable bacteria [13]. Notable methods in this category are polymerase chain reaction (PCR), reverse transcription polymerase chain reaction (RT-PCR), quantitative polymerase chain reaction (qPCR), denaturing gradient gel electrophoresis (DGGE), DNA microarrays, fluorescence in situ hybridization (FISH) and DNA hybridization [14,15,16,17,18,19,20]. The polymerase chain reaction (PCR) is a widely used molecular biology technique that focuses on amplifying specific DNA regions. Its product can then be sequenced and used for bacterial identification by targeting specific bacterial genes or regions and detecting them in the sample if present [14,17]. Among PCR methods, we can distinguish variations in the conventional PCR method, such as asymmetric PCR, nested PCR, multiplex PCR, competitive PCR, qPCR, and RT-PCR [21]. The advanced version, qPCR, quantifies the amount of DNA in a sample, offering insights into bacterial DNA quantity and contamination extent [15,17,22]. Isothermal PCR methods deserve additional attention. Unlike classic PCR methods, they do not require thermocycling but can be performed at constant temperature and simple conditions [23]. In turn, Next-Generation Sequencing (NGS) allows for the sequencing of large numbers of DNA fragments simultaneously. In the context of aerosol samples, NGS can provide a comprehensive overview of all bacterial species present, allowing for detailed analysis of microbial diversity [16]. Another method used to identify bacterial genetic material is the DNA microarray technique, which consists of many nucleic acid sequences on small surfaces that can be used to locate and measure the gene expression [24]. DGGE is a technique used to separate DNA fragments based on their melting behavior. It is often employed to analyze the diversity of microbial communities in aerosol samples by separating PCR-amplified 16S rRNA gene fragments [20]. FISH involves the use of fluorescently labeled nucleic acid probes to specifically bind to target DNA or RNA sequences in microbial cells. It is a direct detection method that can be applied to assess the abundance and spatial distribution of specific microorganisms in aerosol samples [18,25]. Furthermore, DNA hybridization involves the hybridization of DNA probes with complementary sequences in the target microorganisms. It can be used to detect specific microbial species in aerosols [26].

Various nucleic acid amplification techniques are used as molecular methods to evaluate microbial quality in aerosols. One of the amplification techniques applied in aerosol studies focuses on the 16S ribosomal RNA gene, a ubiquitous component in all bacteria. Sequencing this gene enables the identification of various bacterial species within the sample [27]. This method allows for a high-resolution analysis of the bacterial community, aiding in understanding the diversity and composition of aerosolized bacteria. Furthermore, metagenomic analysis entails sequencing all genetic material in a sample, offering insights not only into bacterial species but also into other microorganisms like viruses and fungi that might be present in the aerosols [22]. Advanced molecular techniques, including metagenomic sequencing, are increasingly employed to overcome limitations, providing a more comprehensive understanding of the microbial composition in aerosols, encompassing unculturable species and potential pathogens [28]. Moreover, loop-mediated isothermal amplification is a method for isothermally amplifying DNA, known for its simplicity and rapid detection of specific bacterial DNA [29]. This technique is particularly useful in quickly identifying and quantifying specific bacterial strains in aerosol samples. Additionally, microarray technology enables the simultaneous detection of multiple bacterial species. DNA probes specific to different bacteria are immobilized on a solid surface, and the presence of complementary DNA from the aerosol sample indicates the bacterial species [30]. The use of microarrays enhances the ability to detect and differentiate a broad range of bacterial species in aerosol samples.

Molecular techniques employed for microbial identification in healthcare unit aerosols offer notable advantages, including high precision, sensitivity, and rapid detection of microbial presence, enabling swift responses to potential infections [27,28,29]. The specificity of these methods allows for the accurate differentiation of microbial species, and their multiplexing capabilities enable the simultaneous detection of multiple pathogens [30]. However, the adoption of molecular techniques in healthcare settings faces challenges such as cost constraints, as the equipment and reagents can be expensive. Technical expertise is essential for successful implementation, and the reliance on sophisticated equipment may be a limitation in resource-limited settings [14]. Additionally, the potential for false positives or negatives, influenced by contamination and sample quality, poses considerations. Moreover, the accessibility of molecular diagnostic technologies may be limited in certain healthcare units, particularly in less developed regions, thereby affecting their widespread use for microbial identification in aerosols [14].

The aim of this systematic review was to assess the molecular tests employed for the analysis of aerosols in diverse healthcare settings. The second objective was to investigate whether the implementation of molecular methodologies contributes to enhanced precision in the assessment of bacterial quality within aerosols in healthcare settings. Conducting such a literature review is intended to inspire researchers to pursue additional studies utilizing highly accurate tests to evaluate microbiological hazards in medical facilities. Moreover, a systematic review on this specific topic has not been published to date. This endeavor sought to comprehend the effectiveness, accuracy, and intended applications of selected molecular methods in the examination of bioaerosols.

## 2. Materials and Methods

### 2.1. Focused Question

This systematic review followed the PICO framework as follows: PICO question: In the case of bacteria and fungi included in aerosols (population), what would be the best method to identify and examine (outcome) these microorganisms from a molecular aspect (investigated condition)?

### 2.2. Protocol

**Figure 1:**
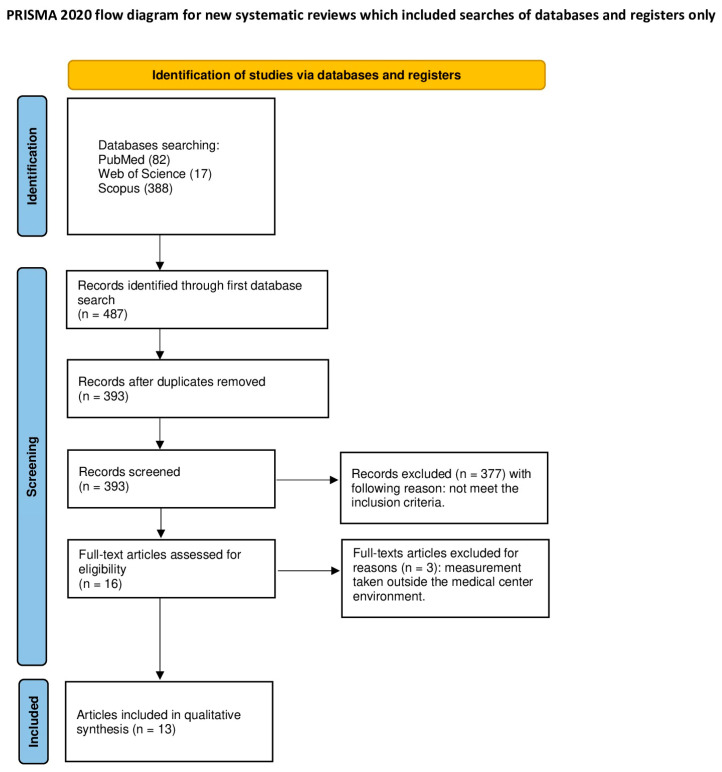
PRISMA 2020 flow diagram.

A detailed presentation and description of selected articles used in this systematic review were outlined in accordance with the PRISMA statement [31]. (Figure 1) The systematic review was registered on the Open Science Framework under the following link: https://osf.io/ysk4t/ (accessed on 2 April 2024).

### 2.3. Eligibility Criteria

The reviewers agreed to include only the articles which met the following criteria listed below:

- Studies that focused on aerosols in healthcare environments (dental clinics, rehabilitation centers, nursing centers, sanatoriums, different hospital areas);
- Presence of bacteria and fungi;
- Application of molecular methods to recognize the bacteria;
- In vivo studies;
- In vitro studies;
- Full-text articles;
- Studies in English.

The exclusion criteria the reviewers agreed upon were as follows:

- Aerosols not focused on aerosols in healthcare environments;
- Studies not including the presence of bacteria and fungi;
- Studies without molecular analysis;
- Non-English studies;
- Systematic review articles;
- Reviews;
- Meta-analysis.

No restrictions were imposed regarding the year of publication.

### 2.4. Information Sources, Search Strategy, and Study Selection

In January 2024, a comprehensive electronic search was carried out in esteemed databases including PubMed, Web of Science (WoS), and Scopus, employing carefully selected keywords such as ((bacteria) OR (virus) OR (fungi)) AND (aerosol) AND ((hospital) OR (healthcare) OR (dental office)) AND ((molecular) OR (PCR) OR (NGS) OR (RNA) OR (DNA) OR (metagenomic) OR (microarray)), following the PRISMA protocol. In PubMed and WoS, the results were refined to titles, authors, and abstracts, while in the Scopus database, the results were narrowed down to titles, authors, and keywords. The search was restricted to studies investigating bacteria and fungi in aerosols using molecular methods. The inclusion criteria encompassed both in vivo and in vitro studies published in English. Studies that did not meet the predefined criteria were excluded. Non-English papers, meta-analyses, and other reviews or systematic reviews were not considered.

### 2.5. Data Collection Process, Data Items

The articles that met the inclusion criteria were extracted by two independent reviewers (J.M., J.K.). The following data were used: first author, year of publication, study design, article title, methods used to examine bacteria and fungi contained in aerosols, and their effectiveness and results. The extracted information was then entered into a standardized Excel file.

### 2.6. Risk of Bias in Individual Studies

At the initial stage of study selection, each reviewer individually checked the titles and abstracts to minimize potential reviewer bias. Cohen’s k test was used as a tool to determine the level of agreement among reviewers [32]. Any disagreement about the inclusion or exclusion of an article in the review was resolved by discussion between the authors.

### 2.7. Quality Assessment

Two reviewers (J.M. and J.K.) independently conducted separate screenings of the included studies to assess the quality of each study. The criteria for evaluating study design, implementation, and analysis included information such as the origin of the aerosol, method of aerosol collection, type of molecular test performed, presence of a control group, sample size calculation, and the number of samples in a group exceeding 10. The studies were assigned scores ranging from 0 to 6 points, with assessments as follows: 0–2 points indicated a high risk, 3–4 points denoted a moderate risk, and 5–6 points indicated a low risk. Any discrepancies in scoring were resolved via discussion until a consensus was reached.

### 2.8. Risk of Bias across Studies

The scores of each study were calculated, and an overall estimated risk of bias (low, moderate, high) was made for each included study, as recommended in the Cochrane Handbook for Systematic Reviews of Interventions [33].

## 3. Results

### 3.1. Study Selection

The PubMed, Scopus, and WoS (Web of Science) database search identified 487 articles potentially applicable for the analysis. After eliminating all duplications, 393 articles were screened. The first screening, which included titles and abstracts, allowed us to exclude 377 articles as they did not focus on the topic of this review. In total, 16 articles were subjected to a full-text analysis, from which 3 were excluded due to not meeting the inclusion criteria (measurements of the exhaled air or non-English papers). In total, 13 articles were qualified for analysis in this systematic review [3,26,34,35,36,37,38,39,40,41,42,43,44].

### 3.2. General Characteristics of the Included Studies

In this systematic review, a total of thirteen articles were included. Most of the studies examined the aerosols present in different hospital areas such as wards, operating rooms, nursing stations, waiting rooms, textile rooms, corridors, etc. [3,26,34,35,36,37,38,39,40,41,42,43]. Among all included studies, one focused on aerosols produced during dental prophylaxis with an ultrasonic scaler [44]. A general characteristic of the included studies has been demonstrated in Table 1.

Most papers included in this review focus on bacteria detection [3,36,37,39,40,41,42]. In addition to examining bacteria, five studies also consider the content of fungi in the air [26,34,35,38,43]. To collect samples for testing, in most cases, specially prepared air samplers were used [3,26,34,35,36,37,38,39,40,42,43]. There were also exceptions where a Petri dish [44] or a specially created microchip [41] was used to collect the aerosol sample.

The predominant molecular technique in the reviewed studies was the polymerase chain reaction (PCR) method, which was applied in eight included studies [3,26,35,37,38,39,42,43]. Chen, P-S et al., in their study, used a modified method of conventional PCR, which was RT-qPCR, which allows direct analysis of viral RNA and enables quantitative analysis of gene expression and RNA quantity [42]. This method focuses on amplifying the 16S rRNA region, selected for its notable conservation [26,34,35,37,38,39,42,43]. Additionally, Habibi et al. employed the 18S rRNA region sequencing in case of fungal identification [38]. Another method of sequencing the genetic material of mushrooms was used by Núñez et al., using ITS sequencing [34]. These methods involve amplifying the DNA chain of bacteria or fungi extracted from samples, enabling their precise identification. The identification process involves comparing the obtained sequences with a comprehensive public database to assess the similarity with sequences present in the gene bank [39]. Notably, this method offers a meticulous approach to determining the taxonomic origin of bacteria or fungi, specifically their classification within a distinct genus and species [3,26,35,37,38,39,42,43].

Retamal-Valdes B et al. [44] employed an alternative molecular methodology, opting for DNA hybridization instead of PCR. This method diverges significantly in its procedural aspects from PCR. The obtained DNA underwent meticulous preparation under specific thermal conditions for an appropriate duration. Subsequently, digoxigenin-labeled whole genomic DNA probes targeting 40 bacterial species were utilized for hybridization. The detection process involved the utilization of anti-digoxigenin antibodies conjugated with alkaline phosphatase, and chemiluminescence detection using a specialized scanner facilitated the identification of specific bacterial species. Analogous to the PCR method, this technique effectively detected the targeted bacterial species [44].

Molecular techniques, such as PCR, offer versatile applications for conducting various molecular tests, as exemplified in a study conducted by Gilbert et al. [43]. In this investigation, PCR was utilized to amplify the extracted DNA, which was subsequently applied to an agarose gel for Denaturing gradient gel electrophoresis (DGGE). This approach uses the PCR, which is loaded in a gel and subjected to a process that allows you to distinguish strips corresponding to different bacterial strains [43]. A different method that involves electrophoresis was used by Wu B et al. [36], where Pulsed Gel Electrophoresis (PFGE) was applied. This method differs from the others in that it is based only on DNA denaturation and not on its amplification. However, this method, despite its different nature, allowed to show a wide genetic polymorphism among examined *E. coli* samples [36]. Other molecular methods used in the qualified studies were SBT [40] and LAMP [41].

Molecular methods are an instrument in detecting genes associated with antibiotic resistance. Wu B. et al. [36], in their study targeting Extended-Spectrum Beta-Lactamase (ESBL)-producing *Escherichia coli*, employed both the conventional culture method for identifying antibiotic resistance and the PCR method for testing the presence of TEM, SHV, and CTX m genes. Unique primers and specific parameters for annealing, extension, and the number of cycles were utilized for each gene. The investigation successfully identified four strains positive for the SHV gene, eight strains positive for the TEM gene, and five strains positive for the CTX m gene. Gene identification proved unattainable via classical methods, which only facilitated Minimum Inhibitory Concentration (MIC) testing to assess resistance to individual antibiotics [36].

Other studies conducted in healthcare facilities such as dental clinics have shown that prevalent bioaerosol pathogens include periopathogens and saliva-borne bacteria, such as *S. mutans*. The molecular genetic profiling of bacteria in generated bioaerosols emphasizes the imperative need for individual protection measures among employees to avoid potential infections [44]. A parallel study by Handorean A. et al. examined airborne bacteria associated with linens used by patients, subsequently cleaned and stored by medical staff. The identified bacteria, including *Staphylococcus*, *Propionibacteria*, *Corynebacteria*, *Lactobacillus*, and *Streptococcus* spp., pose a significant pathogenic risk to employees [37]. A detailed characteristic of the included is presented in Table 2. 

### 3.3. Main Study Outcomes

In all thirteen of the studies included, a consistent application of molecular methodologies was observed for the precise identification of bacterial or fungal genera or exact strains [3,26,34,35,36,37,38,39,40,41,42,43,44]. Additionally, eight investigations integrated traditional microbiological techniques, such as traditional culturing, to allow for comparative analyses with the employed molecular methods [3,26,34,35,36,40,43,44]. In all instances, the classic microbiological culture was not employed with the primary goal of identifying specific bacterial strains. Instead, in most of the presented studies, microbiological culture served the purpose of quantifying the bacterial load. This quantification involved assessing the quantity of bacteria in the compared samples using the unit CFU/m^3^ [3,26,34,35,36,40,43]. This methodology provided a means to evaluate bacterial concentrations across diverse contexts, encompassing various phases of dental procedures, distinct hospital zones, and comparisons between indoor and outdoor settings [3,26,34,35,36,40,43]. While conventional assessments of bacterial diversity relied on culture-based morphology or histopathology, molecular testing emerged as indispensable for precise strain identification and determining the exact species within cultured bacteria [3,34,35,36,37,38,39,40,42,43,44]. The identification of fungal strains was slightly different. In most studies also focusing on fungi, culture was the only form of strain identification [26,34,35,38,43]. Only Núñez et al. and Habibi et al. in their research they used molecular methods to identify fungal strains [34,38].

Furthermore, molecular testing exhibited superiority over classical methods by uncovering a broader spectrum of bacterial species. In the studies by Wu B et al. [36] and Yousefzadeh A et al. [26], bacterial culture served to evaluate antibiotic resistance via the assessment of the inhibition zone and examination of the Minimum Inhibitory Concentration (MIC) values. However, molecular techniques such as PCR offered heightened precision by identifying specific genes responsible for antibiotic resistance, elevating the accuracy of such assessments [26,36]. In addition, bacterial culture was also conducted to prepare material for extracting DNA, which was then subjected to further molecular tests [3,26,34,35,36,40,43,44]. However, in the research conducted by Handorean et al., Habibi et al., Perkins et al., Jiang et al., and Chen et al., the molecular test was performed directly from the collected air samples without the need to perform bacterial culture. Thanks to this, researchers immediately obtained accurate results in the time needed only to perform the molecular method, omitting the time needed for incubation [37,38,39,41,42].

In seven investigations, a predominant molecular methodology was employed, focusing on the PCR amplification of the 16S rRNA gene region [26,34,35,37,38,39,42,43]. In two studies focusing on fungi identification, different kinds of amplification were used: 18S rRNA sequencing and ITS rRNA sequencing [34,38]. The use of this method made it possible to recognize the phylogenetic origin of the studied bacteria contained in the collected aerosol samples. In studies conducted by Wu b et al. and Gilbert Y et al., the 16S rRNA amplification facilitated the electrophoresis analysis of DNA material, providing an alternative means of identifying bacterial species [36,43]. The LAMP method used in the study by Jiang X et al. deserves particular attention. This method, compared to other popular methods, is characterized by a short implementation time, which makes the identification of a given bacterium in a hospital ward much faster than would be possible using another method [41].

Another molecular technique employed in the conducted research within this review was Checkerboard DNA-DNA hybridization [44]. This method, akin to other molecular approaches, was utilized for the identification of bacterial strains present in collected samples. However, the procedure necessitated meticulous preparation, involving the addition of a specific buffer to previously cultivated colonies on plates. Subsequent to this preparatory step, the samples were transferred to Eppendorf tubes before undergoing molecular testing. In contrast to the PCR method, this approach was characterized by increased time requirements and complexity due to the specific demands of sample preparation [44].

### 3.4. Quality Assessment of the Included Studies

Among the articles included in the review, four were appraised as high-quality studies, achieving scores of 5/6 [26,34] or 6/6 [38,40] points. Furthermore, five studies were characterized by a moderate risk of bias, with scores of 4/6 points [35,36,37] or 3/6 points [3,39]. No studies were excluded due to low quality (high risk of bias), as the missing information was deemed non-essential for the thoroughness of the review. The precise risk of bias for each included study is outlined in Table 3.

## 4. Discussion

The aim of this systematic review was to assess the molecular tests employed for the analysis of aerosols in diverse healthcare settings. The second objective was to investigate whether the implementation of molecular methods contributes to enhanced precision in the assessment of bacterial and fungal quality within aerosols in healthcare settings. The investigation delineated that the prevailing molecular method in aerosol quality evaluations was the PCR method [3,26,34,35,36,37,38,39,42,43] using 16S rRNA [3,26,34,35,37,38,39,42,43] or using 18S rRNA sequencing [38] or ITS sequencing [34] in case of fungal analysis. Nevertheless, five of the included studies utilized PFGE [36], DGGE [43], DNA hybridization [44], LAMP [41], or sequence-based typing SBT [40]. As the 16S rRNA sequencing was the most applied type of genetic material analysis, it can be said that it is a powerful and widely used tool for studying bacterial diversity and taxonomy in various environments [26]. Furthermore, 16S rRNA together with 18S rRNA sequencing is advocated as the optimal tool for elucidating the phylogenetic origin of a given bacterium and fungi, respectively, given its conserved nature and minimal susceptibility to alterations [38,45].

In the conducted investigations, molecular methods served as instrumental tools in delineating the precise composition of bacterial and fungal entities within the sampled aerosols [3,26,34,35,36,37,38,39,40,41,42,43,44]. The markedly abbreviated timeframe of these molecular assays (several hours instead of a few days) stands as a notable advantage over conventional techniques, where the cultivation of bacterial cultures necessitates prolonged incubation periods [29,46]. This advantage was strongly emphasized in a study performed by Jiang et al., which shows the advantage of the LAMP method over other molecular methods, indicating a significantly shorter waiting time for the result [41]. Noteworthy is the capacity of these assays to yield comprehensive insights immediately post-sample collection, obviating the requirement for bacterial culture [37,38,39,41,42,47,48]. Such molecular analyses furnish exhaustive details concerning species, genera, and taxonomic diversity, both in the case of bacteria and fungi [37,38,39]. Beyond its efficacy in elucidating bacterial origin and strain-specific identification, molecular testing emerges as a potent tool for the detection of antibiotic resistance—a pivotal facet in comprehending the potential dissemination of antibiotic resistance within a particular strain across the population. The molecular method involves capturing the appropriate sequence in the genetic material that encodes the appropriate protein that confers antibiotic resistance to a given strain [49].

It is essential to acknowledge the ubiquitous presence of aerosols, especially within healthcare environments, where the concentration of pathogenic microorganisms may exceed permissible standards [26,37]. This situation poses a potential risk of exposing healthcare workers and patients to infections associated with airborne bacteria and fungi [37,42]. Dental offices warrant special attention due to the generation of aerosols during various procedures [50]. Ultrasound scaling, a widely employed dental procedure, generates substantial aerosol quantities containing bacteria from the patient’s saliva, gingival sulcus, or pocket, where the most pathogenic oral bacteria reside [51]. The research presented in this article revealed the methods of reducing and eliminating the number of bacteria in aerosols generated during dental procedures. Retamal-Valdes B et al. [44] observed that the use of chlorhexidine or a cetylpyridinium chloride+zinc lactate+sodium fluoride mixture rinse reduces the number of orange complex bacteria, presenting promising results. Ensuring proper disinfection and ventilation in dental rooms is crucial, as aerosols settle in various office areas, facilitating the spread of microorganisms and thereby posing a threat to employees and subsequent patients [44].

In numerous studies, the initial step prior to molecular analysis included culturing bacteria on various agar media [3,26,34,35,36,37,38,40,41,42,43,44,52]. The culture procedure was aimed at quantifying the cultured bacteria in terms of the number of colonies [3,26,34,35,36,40,43] and preliminary identification [40]. Wu B et al. also used bacterial culture to examine antibiotic resistance [36]. Additionally, the culture method was mainly used to determine colony-forming units (CFU), which allowed for the assessment of the total bacterial load in the studies performed [26,34,35,36,40,43,44]. While all studies incorporating bacterial culture later used it for molecular testing, some studies decided to skip the culture step and move directly to molecular testing to increase accuracy and information [37,38,39,41,42]. In the case of identifying fungal strains, the identification was slightly different. In most cases, culture was sufficient to assess the exact species [26,35]. Only in two cases, fungi samples were subjected to a more thorough molecular analysis to determine their species, which precisely described the fungal species [34,38].

This systematic review emphasized the precision achieved in the analysis of bioaerosols via molecular studies. However, it is crucial to acknowledge certain limitations. The features of molecular methods used in research highlight their advantages over classical methods. However, it is important to acknowledge that molecular methods also face certain challenges. One significant challenge is the cost. Wang Y et al. reported that the cost of examining one species using molecular methods can be up to 10 times more expensive than the classic seeding method [53]. Due to the high expenses involved, these methods may not be feasible in economically slower developing countries [46]. Furthermore, some molecular methods still necessitate a previous bacterial culture, thereby extending the time required to obtain results by the duration of the culture time [54]. The advantage here is the identification of ridges, in which molecular identification is not necessary, and therefore performing such a test is cheaper and more accessible [26,35]. Another issue with molecular methods is the presence of mutations. The reliability of these methods is particularly questioned in the context of sequencing the 16S rRNA, which is traditionally considered a conservative part of the genetic material. Recent research indicates that horizontal gene transfer may occur, potentially influencing the 16S rRNA region. This discovery implies that the 16S rRNA’s conservative nature may no longer hold, rendering its use in bacterial identification potentially ineffective [55].

Last but not least, it is worth highlighting that the molecular techniques employed in the included papers in the review also allow for identifying antibiotic resistance genes, focusing on those directly associated with resistance or previously linked to such processes in research or available in databases. By targeting specific genes or genomic regions known to confer resistance, researchers can elucidate the underlying mechanisms driving resistance development in pathogens [56]. Notably, many pathogens regulate their pathogenicity and virulence via a resistance phenotype, sometimes covertly. This phenotype, often governed by complex regulatory networks, can modulate the expression of various genes involved in antibiotic resistance pathways. Researchers can decipher the intricate interplay between resistance mechanisms and microbial fitness via gene expression profiling and functional genomics studies [57]. This phenotype interacts within the genomic network with other genes, leading to bacteriostasis, enhanced fitness, and eventual resistance via a gradual mechanism influenced by time intervals under selective pressure. These processes often entail intricate genetic circuits and transduction chains involving activating or repressing specific genes in response to external stimuli [58]. This interpretation underscores the significance of integrating identification efforts with mechanisms to prevent health risks, including identifying virulent factors. Understanding the complex interplay between antibiotic resistance mechanisms and microbial pathogenesis is essential for developing effective strategies to mitigate the spread of resistant pathogens and safeguard public health [59].

Given that PCR is the predominant research method, there is a lack or scarcity of data obtained from alternative genetic material examination methods, such as FISH, PFGE, DGGE, and DNA hybridization. It should be noted that the above-mentioned molecular methods are being improved and replaced by new, improved methods. A good example is isothermal PCR methods, which do not require large temperature changes during the cycle and can be conducted in constant conditions. Modern non-PCR methods such as Transcription-Based Amplification (TBA) or Strand Displacement Amplification (SDA) also deserve attention. The first of them uses reverse transcriptase to amplify the genetic material, while the second uses DNA polymerase. Their advantage, similar to isothermal processes, is that they carry out processes under constant temperature conditions without the need to use thermocyclers [60]. Many of the modern methods allow you to overcome the limitations associated with conventional methods. An example is Next-Generation Sequencing (NGS), which allows for the analysis of genetic material without the need to perform culture and allows us to focus on longer DNA fragments [61]. To enhance the comprehensive assessment of accuracy and methodological superiority, further investigations are warranted, incorporating a diverse range of modern molecular techniques. These approaches may offer distinct characteristics that can significantly contribute to the nuanced examination of bioaerosols.

## 5. Conclusions

In conclusion, most of the included studies predominantly utilized molecular methods, specifically 16S rRNA gene amplification via PCR for bacterial identification. In the case of fungi identification, in most cases, traditional culture was sufficient to provide information on the identification of a given strain. However, the molecular test also proves to be accurate in this case to confirm the initial identification. This approach consistently identified bacterial and fungal strains, providing precise insights into their origin, genera, and species. Molecular analyses were effective in detecting antibiotic resistance genes. Molecular methods were found to be superior to classical bacterial cultures. They offer more information for strain identification, while the culture mainly provides information on the total bacteria load expressed in CFU/m^3^. However, the culture method seems to be sufficient in the case of fungal identification. Additionally, molecular methods enable the detection of specific gene sequences in the bacterial genetic material, aiding in the identification of genes responsible for antibiotic resistance. Moreover, they require less time to obtain the result—in the case of a culture, it takes several days, and in the case of a molecular test, only a few hours. While molecular testing presents significant advantages over classical microbiological culture, further research is needed to compare various molecular tests and determine the most optimal approach. Overall, this review emphasizes the pivotal role of molecular methods in comprehensively characterizing bioaerosols, understanding their pathogenicity, and assessing health risks for healthcare professionals and patients.

## Figures and Tables

**Table 1:** General characteristics of studies.

Study	Aim of the Study	Material and Methods	Results	Conclusions
Yousefzadeh A. et al. [26]	To establish the concentration and identify bacteria and fungi in bioaerosol present in indoor and outdoor air of a hospital	The air samples were taken from 10 different hospital wards using an Andersen one-stage sampler. Samples were taken from outside the hospital as well. Then, the aerosol was subjected to further culture identification, evaluation of antibiotic resistance, and PCR identification.	The study resulted in the identification of 14 bacterial and 12 fungi species. lowest concentrations of bacteria load were found in the operating room and the highest in the internal men’s ward. The biggest number of fungal species was found in the lung ward. *Staphylococcus hemolyticus* and *Penicillium* were the most abundant. *S. hemolyticus* showed resistance to five types of antibiotics.	The air quality in some wards exceeded the WHO standards despite good ventilation systems.
Núñez, A. et al. [34]	Comparison of hospital indoor and outdoor aerosols at two different seasons to evaluate the influence of the urban atmosphere, determine the presence of pathogens, and to evaluate the effectiveness of ventilation.	A total of 45 samples were collected in summer and wintertime from indoors and outdoors of the hospital. The samples were taken using the Petri dishes and then subjected to DNA extraction and sequencing- targeting the bacterial and fungal DNA.	The results showed a striking microbial variability associated with the year season. In summer, the predominant bacterial and fungal taxa were Proteobacteria and Ascomycota, while in winter, Basidiomycota showed the highest abundance. Opening the window to ventilate the room showed minor variations in air composition.	The indoor air is highly influenced by outdoor conditions. Changes in bacterial composition depending on the season
Nimra, A et al. [35]	To explore microbial diversity in differentially ventilated orthopedic wards (OW) and emergency rooms (OER) via the application of molecular techniques and biochemical testing.	The samples were taken from two different hospitals- one with central air conditioning (group I) and the other with non-central air conditioning (group II). Both the indoor and outdoor air was sampled. The samples were subjected to culture identification and quantification and phylogenic analysis involving 16S rRNA sequencing.	The molecular analysis observed the most abundant species, such as *B. cereus, B. subtilis, C. perfringens, E. coli, K. pneumoniae, S. aureus* and *M. luteus.* The indoor bacteria load was lower in the case of hospitals with central air-conditioning. The fungal analysis showed *A.flavus* and *A. niger* as the most abundant species in both OW, *Penicillium,* and *A. niger* in OER in group I and *A. flavusand A. niger* in group II.	Effective air circulation is crucial for maintaining air cleanliness in healthcare facilities. Moreover, molecular testing allowed for the detection of specific bacterial strains. In the case of bacterial strains, identification based on culture alone was sufficient to identify the most common strains.
Wu B et al. [36]	The aim of the study was to examine the load of *E. coli* in the air located in different parts of the hospital ward and to check its genetic similarity in terms of antibiotic resistance.	Samples from different ward environments were collected using Andersen-6 impact type and RCS centrifugal air microbial samplers. Air samples were also taken from the corridor 5 and 10 m far from the ward. Collected bacteria were cultured on media and subjected to PFGE testing. The PCR method was employed to examine antibiotic-resistance genes, specifically TEM, SHV, and CTX-M.	The concentration of airborne aerobic bacteria varied between each ward. *E. coli* was present in each ward and showed the highest concentration in ward A. The results showed a presence of *E. coli* also in spaces outside the ward. The tests allowed us to determine the antibiotic resistance of the strain found in each ward and after.	This study allowed us to isolate and identify the *E. coli* strains in different parts of the hospital. The study also assesses the antimicrobial resistance profiles. Molecular PFGE showed that *E. coli* can be spread through the air into parts outside of the ward possessing a potential threat to the health of patients.
Handorean A et al. [37]	Examination of airborne bacteria contained in the air in hospital textile storage rooms during the period between its storage and removal for laundry.	The aerosol was collected from different textile rooms, filtered by special filters, and subjected to PCR testing.	The research did not show any differences in the composition of the air when bed linen was delivered and taken away. The PCR technique allowed us to distinguish the bacteria found in the samples.	Handling hospital textiles may expose health workers to infections.
Habibi, N. et al. [38]	The aim of this study was to investigate indoor aerosols in two hospitals and to identify bacterial and fungal identification.	The samples were collected from two hospitals involved in COVID-19 treatment and from non-hospitalized settings. The samples were then subjected to 16S rDNA and ITS sequencing and PCR amplification.	The molecular investigation allowed us to determine the exact taxonomic composition of both bacterial and fungal communities. The bacteria composition differed between hospitals and non-hospital facilities while the fungal community was more homogenous.	The molecular methods were effective in the identification of bacterial species present in examined facilities.
Angenent LT et al. [3]	General molecular analysis of bioaerosol in public pools and public health facilities.	Samples of the pool air, the air just above the pool, pool water and the air from the outside of the pool building were collected. Then, they were subjected to colony and microscopy count. Later, the DNA sequencing was conducted (PCR).	Regardless of the sample, bacterial counts were significantly higher than in routine samples. Moreover, you managed to determine the phylogenetic origin of the bacteria contained in the samples. A high percentage of bacteria turned out to be *M. avium* clones.	The results of the study confirm the transmission of bacteria, which means the transmission of diseases. Furthermore, the protocols responsible for removing potential pathogens are clearly inadequate.
Perkins SD et al. [39]	To investigate if shower stalls in a stem cell transplant unit in a hospital can be a source of pathogens by analyzing the bacterial load, colony count, and bacterial DNA.	Samples were collected from the shower stall in a hospital with a stem cell transplant unit. Samples were collected for 6 days from the shower stall with and without membrane-integrated showerheads prior to turning on the shower, and then shower aerosol samples were collected from the same location as the water was running. Lastly, one liter of shower water was collected. Then, the samples were subjected to quantitative and molecular (PCR) analysis.	There were more pathogenic bacteria detected in collected water than in shower aerosol samples. Most notable was the presence of *M. mucogenicum* in water and *P. aeruginosa* in aerosol samples. However, in the samples collected from the shower stall where the membrane-integrated showerhead was installed, a reduced number of bacteria was observed.	Using membrane-integrated showerheads in shower stalls in a stem cell transplant unit can help to reduce the microbial load in water and surrounding aerosol, which can prevent infections in patient populations.
Montagna, M.T et al. [40]	To compare the *Legionella pneumophila* serogroups present in water and air samples collected from 10 healthcare facilities.	The hot water from the tap was sampled as well as the air from around the tap. Colonies classified as *Legionella* were subjected to a latex agglutination test with polyvalent antisera. Then, a molecular analysis was conducted on 17 strains.	The most prevalent *Lgn* serogroup was serogroup 6, followed by 9, 1, 7, and 12. In water samples, only *Lgn* serogroup 6 was detected.	The study does not show a correlation between water and surrounding air contamination. However, it shows the significance of serotyping, enabling a more precise evaluation of the dissemination of Legionella serogroups in the environment.
Jiang, X et el. [41]	The use of microfluidic chip for the rapid detection of *Staphylococcus aureus.*	An S. aureus culture was diluted to different concentrations of bioaerosol and then captured by a specially fabricated microchip. A clinical airborne sample was also collected from six different settings of a hospital, including surgery rooms, ICU, surgical wards, outpatients’ service hall and doctor’s office. The samples were then subjected to a LAMP reaction.	Fluorescent detection was used to evaluate the concentrations of S. aureus. The in vitro samples showed high fluorescence when many bacterial cells were present. Fluorescence decreased as the number of cells in the sample decreased. In the case of clinical samples, the fluorescence was very low, which means that S. aureus concentrations did not exceed the detection limit.	The presented technique is a promising method that can be applied in disease control due to significant time savings compared to other methods.
Chen, P.-S et al. [42]	The use of RT-PCR to assess the concentration profiles of airborne *M. tuberculosis* in a hospital, encompassing both patient-related and non-patient-related areas.	In total, 24 samples were taken from non-tuberculosis areas and 34 from tuberculosis areas. The DNA was extracted from the samples, and M. tuberculosis was detected using RT-qPCR.	The TB area M. tuberculosis concentrations were significantly higher than in non-TB areas. The highest airborne M. tuberculosis was detected in waiting and consulting rooms, emergency departments and medical wards.	The study emphasizes the significance of identifying high-risk areas and implementing tuberculosis control strategies to protect healthcare workers and patients within hospital environments.
Gilbert, Y et al. [43]	Analysis of bacterial diversity in a newly occupied hospital and assessment of whether sinks may be a potential reservoir of bioaerosols and a reservoir of opportunistic bacteria and antibiotic-resistant genes	The sink biofilm samples and air samples were taken from empty patients’ rooms. Extra cubic meters of air were collected to assess the presence of antibiotic-resistance genes. The tap water was also sampled. The collected material was subjected to molecular identification using 16S rRNA amplification and PCR. The biodiversity was analyzed using DGGE.	The results showed slightly higher concentrations of airborne microorganisms in patients’ rooms than in control rooms. The predominant bacteria species were S. epidermidis, *S. hominis*, *Bacillus* sp., and *M. luteus*. The most prevalent fungal species were *Cladosporium* sp. and *Penicillium* sp. The molecular methods showed the presence of many antibiotic-resistance genes.	Actions should be taken to minimize the presence of opportunistic pathogens in the air and water to avoid potential infections of patients present on the hospital premises.
Retamal-Valdes B et al. [44]	To evaluate the effect of a pre-procedural mouthwash containing sodium fluoride (F), zinc lactate (Zn), or cetylpyridinium chloride (CPC).	Three plates were placed on a support board attached to the reflector, on the bracket tray, and on the office bench to test the dental office environment. Four groups of 15 people were invited to the study. The first group rinsed the mouth with the mixture of CPC+Zn+F, the second group rinsed with water, the third group with chlorhexidine, and the fourth group did not rinse the mouth. Then, each patient was seated on the chair, and three new plates were placed on the support board, on the patient’s chest, and on the dentist’s forehead. The dentist processes the full-mouth dental prophylaxis. Then, the plates were incubated and analyzed using DNA hybridization.	No bacteria growth was detected on the plates located in the office before the procedure. The bacterial count was significantly lower in patients from group number 1 than group number 2, 3, or 4. When all locations were considered together, the results showed that the number of bacteria in the case of CPC+Zn+F or chlorhexidine rinse was lower than in patients who rinsed only with water or did not rinse at all. The DNA-DNA hybridization showed the presence of 40 subgingival species.	The mouthwash containing CPC+Zn+F was an effective way to reduce bacterial species present in oral aerosols during prophylaxis using ultrasonic instruments.

**Table 2:** Microbiological culture and molecular methods in the evaluation of aerosol quality.

Authors	Aerosol Collection Method	Place of Aerosol Collection	Microbiological Culture Method	Molecular Method	Results (Microbiological Culture)	Results (Molecular Analysis)
Yousefzadeh A et al. [26]	-Andersen one-stage sampler -Bacteria collecting time: 20 min -Fungi collecting time: 5 min	-Inside the hospital (men’s ward, women’s ward, lung, neurology, infectious and burns wards, ICU, operating and emergency room) -Outside the hospital	-Collected samples were incubated for ~48 h in 35–37 °C -Biochemical tests for microscopic and morphologic examination -The Kirby–Bauer disk diffusion method for antibiotic resistance evaluation	-DNA extraction by phenol–chloroform method -PCR method (16S rRNA amplification)	-Bacteria: the emergency room showed the highest concentrations; bacteria concentrations depended on many factors like bed number or ventilation; the most abundant species: *Staphylococcus hemolyticus* -Fungi: the highest concentration was detected on the long ward, the lowest in the operating room; the most abundant species *Penicillium* and *cranosporium* and yeasts and scopolariopsis	-Performed on two samples that showed the highest abundance and resistance -Sample one: resistance to ciprofloxacin, gentamicin, azithromycin, amoxicillin, cefixime -Sample two: resistance to gentamicin, azithromycin, amoxicillin, cefixime
Núñez, A et al. [34]	-Impactor-type device (DUO SAS Super 360 (VWR)) -Air sampling between 9:00 a.m. and 14:00 p.m.	-Inside de hospital (a room next to the inpatient wing on the 4th floor) -Outside the hospital on the roof of the hospital wing)	-Petri dishes with Nutrient Agar supplemented with amphotericin to prevent fungal growth -Incubation of collected samples for 3 days at 35 °C	-DNA extraction using DNeasy Powersoil Kit (Quiagen) -16S rRNA amplification for bacteria -ITS sequencing for fungi	-Bacteria concentration indoors was lower than outdoors	-The bacteria composition differs between summer and winter -Opening and closing the window had an influence on air composition inside the hospital -The prodominant bacteria species: *Sphingomonas*, *Streptomyces*, *Massilia*, *Hymenobacter* and *Methylobacterium-Methylorubrum* -The predominant fungi species: *Cladosporium*, *Alternaria*, *Filobasidium* and *Penicillium*
Nimra, A et al. [35]	-Volumetric pump sampler -Sampling time 15 min	-Orthopedic wards (OW) and emergency rooms (OER) -Two hospitals- one centrally air-conditioned and the other without central air-conditioning.	-Nutrient agar plates: incubated for 24 h at 37 °C -Sabouraud dextrose agar: incubated for 6 to 7 days at 28 °C.	-PCR (16S rRNA amplification) -Flow cytometry analysis	-Bacteria: the culture study showed a lower bacterial count in OW and OER in an air-conditioned hospital -Fungi: the fungal count showed no significant difference between the two OW, but in OER in a non-air conditioned hospital, the fungal load was higher; the examination allowed to identify: *A. flavus* and *A. Niger* in both OW and higher amounts of *Penicillium* and *A. niger* in OER in air-conditioned hospital compared to non-air conditioned hospital where *A. flavus* and *A. Niger* were more abundant.	-The most observed colonies were identified by PCR, which showed Gram(+) rods: *Bacillus cereus*, *Bacillus subtilis*, and *Clostridium perfringens*, Gram(−) rods: *Escherichia coli* and *Klebsiella pneumonia*, and the Gram(+) cocci *Staphylococcus aureus* and *Micrococcus luteus*
Wu B et al. [36]	-Andersen-6 impact-type sampler -RCS centrifugal air microbial -Sampling time 1 to 5 min	-Samples were taken from 3 different wards (wards A, B, and C) from 3 different points -Samples were also taken from the corridors 5 m and 10 m away from the wards	-Agar supplemented with 5% ram blood and 1% glucose. -Incubation for 24–48 h at 37 °C -Antibiotic resistance test by determining the zone of bacterial growth inhibition.	-Pulsed-field gel electrophoresis (PFGE)	-The highest bacterial count was observed in Ward A, while the lowest count was found in Ward B, with a notable difference. -This trend was similarly reflected in the specific concentration of *E. coli.* -The *E. coli* isolated from ward A showed a complete resistance to ampicillin.	-The PFGE analysis revealed a genetic polymorphism among the isolated species. -The test facilitated the comparison of *E. coli* strains obtained from three distinct wards. -Samples collected from the corridor outside the ward exhibited high similarity to ward samples, suggesting the possible airborne transmission beyond the ward.
Handorean A et al. [37]	-Liquid impingers modified for ultra-clean DNA recovery -Liquid impingers modified for ultra-clean DNA recovery -Liquid impingers modified for ultra-clean DNA recovery -Sampling time: 48 h periods in February (prior to patients’ occupation) and June (initial patients’ occupation)	-Hospital holding textile rooms	-	-PCR (16S rRNA sequencing)	-	Higher concentrations of *Staphylococcus*, *Propionibacteria*, *Corynebacteria*, *Lactobacillus*, and *Streptococcus* spp. were found, significantly surpassing levels in patient rooms with clean bedding. Seasonal variations influenced bacterial content, with Propionibacterium species dominating in winter while other species remained relatively stable.
Habibi, N. et al. [38]	-A customized sampler -Sampling time: 2 h	-Hospital I: near the main entrance, reception, pediatric consualty, central laboratories, pharmacy, and COVID ward -Hospital II: COVID isolation areas and ward, virology, and cytology laboratories -Non-hospitalized setting	-	-Bacteria: PCR (16S rRNA amplification) -Fungi: PCR (18S rRNA amplification)	-	-The molecular testing provided the information about fungal and bacteria taxa -The tests allowed to identify of the most abundant species present in each examined place in both hospitals and non-hospitalized settings
Angenent LT et al. [3]	-γ-radiated filter cassette -Swirling aerosol collectors (SACs) -Sampling time 1h or 2 h 40 min depending on the season.	-Hospital therapy pool with independent ventilation system -Air from the outside of the hospital	-Tryptic soy agar -Incubated for 2–3 weeks at 37 °C.	-PCR -Cloning -Restriction fragment length polymorphism (RFLP) -Sequencing	-The number of colonies in all samples was always higher inside the therapy pool area than outside. -The number of colonies did not represent the real bacterial load in the samples; therefore, the test requires more specialized methods than the culture method.	-Seventy-seven mycobacterial rRNA genes were identified in the air samples, with some closely related to pathogenic species like *M. avium*. -*Prevotella melaninogenica*, associated with vaginitis, periodontal disease, and sinusitis, was detected in the air. -Additionally, *Staphylococcus* spp. and *Streptococcus* spp. or *Alloiococcus otitis*, responsible for ear infections, were present in the pool air.
Perkins SD et al. [39]	-Swirling aerosol collector -Sampling time: 90 min	-Hospital shower prior to turning on the water and while the shower was turned on -The samples were taken while using a conventional shower head and with a Pall-Aquasafe water filter -Shower water	-Microbiological analysis was carried out only for the sample of collected water, which is irrelevant to this review.	-PCR (16S rRNA amplification)	-	-The DNA concentration in the winter samples and in all samples collected under the water filter conditions were too low for successful sequencing. -Samples collected under ordinary showerhead conditions detected the presence of bacteria from Proteobacteria genera. -Detailed phylogenetic analysis detected 444 operational taxonomic units. -The most pathogenic bacteria detected in air samples was *P. aeruginosa*.
Montagna, M.T et al. [40]	-Surface Air System -Petri dishes -Total sampling time: 8 h	-Air from around the tap from ten hospitals	-Petri dishes with Glycine-Vancomycin-Polymyxin-Cycloheximide medium	-Sequence-based typing (SBT)	-As the *Legionella pneumophilia* 6 serotype was the most abundant, the culture method allowed to establish the bacteria concentrations in collected samples (CFU/L)	-The SBT investigated the allelic profiles of the detected *Legionella* strains. -A more exact spread of *Lgn* serotypes could be established
Jiang, X et al. [41]	-A fabricated microfluid chip	-Cultured *S. aureus* -Intensive care unit, surgery room, emergency room, surgical ward, outpatient service hall and doctor’s office	-	-Loop-mediated isothermal amplification (LAMP)	-	-The LAMP results of the samples from the hospital were negative because the collected number of bacteria was lower than the limit of detection (LOD)
Chen, P.-S et al. [42]	-Nuclepore filter -Sampling time: 8 h (4 h in consulting rooms)	-Tuberculosis (TB) positive patients’ areas -Suspected TB patients’ areas -Non-TB patients’ areas	-	-RT-qPCR	-	-The *Mycobacterium tuberculosis* concentrations in TB-positive areas were significantly higher than in non-TB areas. -The increased concentrations of *M. tuberculosis* implicate a high risk of nosominal infections in health workers or other patients
Gilbert, Y et al. [43]	-Six stage Andersen impactor -Bacteria sampling time: 20 min -Fungi sampling time: 5 min	-Ten pulmonology ward rooms with recently gone patients -Control room	-Bacteria: tryptic soy agar and blood agar incubated for 48 h at 25 °C and 37 °C, respectively -Fungi: rose Bengal agar, incubated for 7 days at 25 °C	-PCR (16s rRNA sequencing) -*Denaturing gradient gel electrophoresis (DGGE)*	-Bacteria: the concentrations in hospital rooms did not show a significant difference compared to the concentrations in the control room -Fungi: the fungal concentrations were significantly higher than in control samples	-Dominant bacteria identified: *Staphylococcus epidermidis*, *Staphylococcus. hominis*, *Bacillus* spp. and *Micrococcus luteus* -Dominant fungi identified: *Cladosporium* sp. and *Penicillium* sp. -The molecular tests detected antibiotic resistance in many strains, like vancomycin resistance in *S. paucimobilis*
Retamal-Valdes B et al. [44]	-Agar plates	-Before the dental procedure: support board attached to the reflector, the bracket tray, and the office bench -During the dental procedure: the support board, volunteer’s chest, and clinician’s forehead.	-Tryptic Soy Agar with Yeast Extract enriched with 5% menadione, 5% sheep blood, and 1% N-Acetylmuramic acid -Incubated for 72 h at 37 °C.	-DNA hybridization	-No bacterial growth was detected in the samples collected from the dental office before the procedure. -The colony-forming units (CFU) of the bacteria gathered from the patient’s chest and operator’s forehead were significantly lower in groups who rinsed with CPC+Zn+F and CHX compared to groups with water or no rinse.	-The DNA hybridization technique identified 40 species of subgingival bacteria species -In groups rinsing with CPC+Zn+F or CHX, the aerosol generated during full-mouth dental prophylaxis contained a smaller quantity of orange complex bacteria compared to the group rinsing with water or not rinsing.

**Table 3:** The results of the quality assessment and risk of bias across the studies.

Criteria	Study												
	Yousefzadeh A et al. [26]	Núñez, A et al. [34]	Nimra, A et al. [35]	Wu B et al. [36]	Handorean A et al. [37]	Habibi, N et al. [38]	Angenent LT et al. [3]	Perkins SD et al. [39]	Montagna, M.T et al. [40]	Jiang, X et al. [41]	Chen, P.-S et al. [42]	Gilbert, Y et al. [43]	Retamal-Valdes B. et al. [44]
Origin of the aerosol	1	1	1	1	1	1	1	1	1	1	1	1	1
Method of collecting the aerosol	1	1	1	1	1	1	1	1	1	1	1	1	1
Method and type of molecular test performed	1	1	1	1	1	1	1	1	1	1	1	1	1
Presence of a control group	0	1	0	0	1	0	0	0	0	1	1	0	1
Sample size calculation	1	1	1	1	0	1	0	0	0	0	1	0	1
Number of samples in the group of more than 10	0	0	0	0	0	0	0	0	0	0	0	0	1
Total	4	5	4	4	4	4	3	3	3	4	5	3	6
Risk of bias	moderate	low	moderate	moderate	moderate	moderate	moderate	moderate	moderate	moderate	low	moderate	low

## Data Availability

Availability of supporting data—the datasets used and/or analyzed during the current study are available from the corresponding author upon reasonable request.
